# Quantification of epicardial adipose tissue in morbidly obese patients before and after bariatric surgery

**DOI:** 10.1186/1532-429X-16-S1-P281

**Published:** 2014-01-16

**Authors:** María Luaces, Victoria Cachofeiro, Jorge Cabezudo Pedrazo, Alfonso Antequera-Pérez, Manuel Medina-García, Alejandro García- Muñoz, Leopoldo Pérez de Isla

**Affiliations:** 1 Hospital Clínico San Carlos, Madrid, Spain; 2 Physiology, Universidad Complutense, Madrid, Spain; 3 Hospital Universitario de Fuenlabrada, Fuenlabrada, Spain

## Background

Epicardial adipose tissue is a highly complex endocrine organ that generates various molecules with profound local and systemic effects that can play a role in obesity- related heart damage. Bariatric surgery is a last-step therapeutic option to ameliorate obesity. Objective: To compare the amount of epicardial adipose tissue, as well as anatomic and functional features of the heart, and metabolic profile of morbidly obese patients referred for bariatric surgery

## Methods

**Figure 1:**
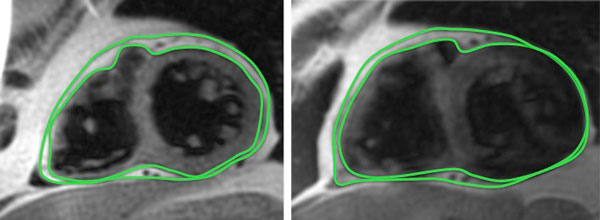
**Short - axis dark- blood images showing the delineation of epicardial fat before (left) and after (right) bariatric surgery**.

Morbidly obese patients accepted for bariatric surgery were prospectively included. In each case, CMR was performed with a 1.5 equipment previous to surgery and with a 3.T magnet 1 year after surgery. The epicardial adipose tissue (EAT) was assessed from a dark-blood prepared multislice turbo-spin echo pulse sequence to obtain short- axis images. The amount of EAT was calculated by using the modified Simpson's rule. The contours of epicardial adipose tissue were outlined covering the entire right and left ventricle (the figure 1). The EAT volume was obtained after the data summation of all slices. For LV structure and function, a conventional SSFPB protocol including short and long-axis slices was acquired. LV mass was calculated using a dedicated software. Pre and postoperative levels of glucose, triglyceride and cholesterol were compared.

## Results

31 patients reached 1 year follow -up. Epicardial adipose tissue decreased by 31%, and LV mass decreased by 26% (the table 1). Glucose and triglyceride levels showed also significant reductions

## Conclusions

CMR is a robust technique to quantify epicardial adipose tissue in morbidly obese patients. The amount of epicardial adipose tissue shows a significant reduction in morbidly obese patients one year after bariatric surgery. This decrease is accompanied by substantial amelioration in metabolic control.

## Funding

Fondo de Investigaciones Sanitarias. Instituto de Salud Carlos III. Ministry of Economy and Competitiveness of Spain. (PI09/0871 and PI09/2428).

**Table 1:** 

n = 31	PRE	POST	p value
BMI (kg/m2)	46.42 ± 5.61	31.12 ± 5.52	< 0.001

Epicardial adipose tissue (mm3)	79.8 ± 30	55.4 ± 23.6	< 0.001

LV mass (g)	113.4 ± 24.9	95.4 ± 26.17	< 0.001

LVEDV (ml)	153.86 ± 30.17	156.55 ± 19.9	0.60

LVESV (ml)	56.62 ± 16.28	56 ± 19.9	0.81

LV SV (ml)	97.88 ± 24.29	101.02 ± 20.8	0.52

LVEF (%)	63.72 ± 10.1	64.7 ± 5.56	0.54

Glucose (g/dL)	103.8 ± 6.26	83.9 ± 3.36	0.007

Triglyceride (mg/dL)	136.50 ± 16.13	93.8 ± 9.85	0.022

Data are expressed as mean ± standard deviation.

